# Does the experience of the bedside assistant effect the results of robotic surgeons in the learning curve of robot assisted radical prostatectomy?

**DOI:** 10.1590/S1677-5538.IBJU.2018.0184

**Published:** 2019

**Authors:** Haci Ibrahim Cimen, Yavuz Tarik Atik, Serkan Altinova, Oztug Adsan, Mevlana Derya Balbay

**Affiliations:** 1 Department of Urology, Sakarya University, School of Medicine, Sakarya, Turkey;; 2 Ankara Ataturk Training and Research Hospital, Ankara, Turkey;; 3 American Hospital, Istanbul, Turkey

**Keywords:** Prostatic Neoplasms, Robotics, Prostatectomy

## Abstract

**Introduction:**

The success of the robot assisted radical prostatectomy (RARP) procedures depend on a successful team, however the literature focuses on the performance of a console surgeon. The aim of this study was to evaluate surgical outcomes of the surgeons during the learning curve in relation to the bedside assistant's experience level during RARP.

**Materials and Methods:**

We retrospectively reviewed two non - laparoscopic, beginner robotic surgeon's cases, and we divided the patients into two groups. The first surgeon completed the operations on 20 patients with a beginner bedside assistant in February - May 2009 (Group-1). The second surgeon completed operations on 16 patients with an experienced (at least 150 cases) bedside assistant in February 2015 - December 2015 (Group-2). The collected data included age, prostate volume, prostate specific antigen (PSA), estimated blood loss, complications and percent of positive surgical margins. In addition, the elapsed time for trocar insertion, robot docking, console surgery, specimen extraction and total anesthesia time were measured separately.

**Results:**

There were no significant differences between the groups in terms of age, comorbidity, prostate volume, PSA value, preoperative Gleason score, number of positive cores, postoperative Gleason score, pathological grade, protection rate of neurovascular bundles, surgical margin positivity, postoperative complications, length of hospital stay, or estimated blood loss. The robot docking, trocar placement, console surgery, anesthesia and specimen extraction times were significantly shorter in group 2 than they were in group 1 (17.75 ± 3.53 min vs. 30.20 ± 7.54 min, p ≤ 0.001; 9.63 ± 2.71 min vs. 14.40 ± 4.52 min, p = 0.001; 189.06 ± 27.70 min vs. 244.95 ± 80.58 min, p = 0.01; 230.94 ± 30.83 min vs. 306.75 ± 87.96 min, p = 0.002; 10.19 ± 2.54 min vs. 17.55 ± 8.79 min, p = 0.002; respectively).

**Conclusion:**

Although the bedside assistant's experience in RARP does not appear to influence the robotic surgeon's oncological outcomes during the learning curve, it may reduce the potential complications by shortening the total operation time.

## INTRODUCTION

Prostate cancer (PCa) is the most common non - cutaneous malignancy and remains the second leading cause of death from cancer in men (1). Radical prostatectomy (RP) is offered as the first line treatment modality for patients with clinically localized PCa and a life expectancy of at least 10 years (2). Open RP has long been the gold standard procedure (3), but laparoscopic and later, robotic prostatectomies have been introduced with the expectation of minimizing peri - and postoperative complications (4-6). Robotic surgery provides three - dimensional magnification and tools with seven degrees of freedom that can duplicate hand movements. Therefore, non - laparoscopic surgeons especially tend to prefer robotic systems, as they reduce the difficulty involved in performing complex laparoscopic procedures (7).

Surgeons skilled in robotic manipulation are physically separated from the patient, resulting in the absence of tactile feedback; such surgeons need a mediator to complete the operation. Although the role of the assistant in robot assisted radical prostatectomy (RARP) was previously described (8), the urologic literature focuses on the performance of the console surgeon (9). However, it is well known that successful RARP depends on a successful team, and the bedside assistant represents a major part of such success (10). RARP is not a solo surgery, as teamwork is crucial for these operations. Therefore, it is important to focus on all members of the team, and not just the primary surgeon. To address the gap in the literature, we want to call attention to the lack of focus on the bedside assistant; thus, in this study, we evaluate the outcomes for robotic surgeons who are in the learning curve for this surgery according to the bedside assistant's level of experience.

## MATERIALS AND METHODS

This study was approved by the local ethics committee. We retrospectively reviewed two non - laparoscopic, beginner robotic surgeon's first series of cases, and divided the patients into two groups. The first surgeon (MDB) completed 20 consecutive operations in February 2009 - May 2009, with a beginner bedside assistant (Group-1). The second surgeon (OA) completed 16 consecutive operations in February 2015 - December 2015, with an experienced (at least 150 cases) bedside assistant (Group-2).

Both surgeons completed the operations with the same transperitoneal posterior approach. None of the patients underwent lymph node dissection according to the Partin nomograms (11). The data collected included: age, prostate volume, prostate specific antigen (PSA), estimated blood loss, complications and percent of positive surgical margins. In addition, elapsed time for trocar insertion, robot docking, console surgery, specimen extraction and total anesthesia time were measured separately. The trocar insertion time was defined as the time from the first attempt to insert the Veress needle through abdominal cavity to the final trocar placement. The robot docking time was defined as the time from the first attempt to insert the Veress needle through abdominal cavity to the connection of the robotic arms to trocars and installation of the robotic instruments and camera. The console surgery time was measured from when the console surgeon began to handle the robot to when the instruments were removed from the patient. The specimen extraction time was defined as the measured time from undocking the patient cart to closing the port sites. Finally, the total anesthesia time referred to the time from induction to arrival at the recovery room.

Statistical analyses were performed using SPSS 20.0 (IBM, NY, USA) statistical program. The normal distribution suitability of the variables was investigated using the one - sample Kolmogorov - Smirnov test. Variables with normal distribution were shown by mean and standard deviation (mean ± SD), whereas variables with non - normal distribution were shown by median (min - max). The comparative analysis of variables was applied using the independent samples t - test or Mann Whitney U test for quantitative parameters and Chi - square or Kruskal - Wallis test for categorical parameters. The level of significance for all analyses was set at 5%.

## RESULTS

Group 1 consisted of 20 patients, while group 2 consisted of 16 patients. There were no significant differences between groups in terms of age, comorbidity, prostate volume, PSA value, preoperative Gleason score, number of positive cores, postoperative Gleason score, pathological grade, protection rate of neurovascular bundles, surgical margin positivity, postoperative complications, length of hospital stay, and estimated blood loss (Table-1). The trocar placement time was statistically shorter in group 2 than it was in group 1 (9.63 ± 2.71 min vs. 14.40 ± 4.52 min, p = 0.001, respectively). Furthermore, the robotic docking time was significantly shorter in group 2 than it was in group 1 (17.75 ± 3.53 min vs. 30.20 ± 7.54 min, p ≤ 0.001, respectively; Table-2). The console surgery, anesthesia and specimen extraction times were also significantly lower in group 2 than they were in group 1 (189.06 ± 27.70 min vs. 244.95 ± 80.58 min, p = 0.001; 230.94 ± 30.83 min vs. 306.75 ± 87.96 min, p = 0.002; 10.19 ± 2.54 min vs. 17.55 ± 8.79 min, p = 0.002; respectively).

**Table 1:** Baseline and pathological characteristics.

		Group 1 (n=20)	Group 2 (n=16)	p value
Age (years) (mean ± SD)		63.00 ± 6.09	63.38 ± 6.85	0.86*
CCI, median (IQR)		0 (0-1)	0 (0-1)	0.815**
Prostate volume (gr) (mean ± SD)		46.65 ± 12.14	48.38 ± 15.78	0.71*
PSA (ng/dL) (min-max)		5.8 (4.5-39)	6.62 (1.47-26.5)	0.82**
**Preop. Gleason score (%)**				
	6	14 (70)	14 (87.5)	
	7	4 (20)	2 (12.5)	
	8-10	2 (10)	0	0.26***
Positive core number		3.45 ± 2.40	3.88 ± 2.55	0.61*
**Clinical stage (%)**				
	T1c	16 (80)	9 (56.25)	
	T2a	4 (20)	3 (18.75)	
	T2b	0	2 (12.50)	
	T2c	0	2 (12.50)	0.16***
**Postop. Gleason score (%)**				
	6	13 (65)	10 (62.5)	
	7	6 (30)	5 (31.25)	
	8-10	1 (5)	1 (6.25)	0.88***
**Pathological stage (%)**				
	T2a	2 (10)	2 (12.5)	
	T2b	2 (10)	2 (12.5)	
	T2c	10 (50)	7 (43.75)	
	T3a	6 (30)	4 (25.00)	
	T3b	0	1 (6.75)	0.08***
Protection of neurovascular bundle (%)		18 (90)	15 (93.75)	0.84***
Positive surgical margin (%)		3 (15)	4 (25)	0.68****

**CCI** = Charlson Comorbidity Index;

* = Independent samples t test;

** = Mann-Whitney U test;

*** = Kruskal Wallis test;

**** = Chi-square test

**Table 2:** Perioperative data.

	Group 1 (n=20)	Group 2 (n=16)	p value
Trocar insertion time, min	14.40 ± 4.52	9.63 ± 2.71	0.001*
Robot docking time, min	30.20 ± 7.54	17.75 ± 3.53	<0.001*
Console surgery time, min	244.95 ± 80.58	189.06 ± 27.70	0.01*
Anesthesia time, min	306.75 ± 87.96	230.94 ± 30.83	0.002*
Specimen extraction time, min	17.55 ± 8.79	10.19 ± 2.54	0.002**
Estimated blood loss, mL	268 ± 236.57	281.25 ± 178.02	0.85*
Median length of hospital stay (days) (min-max)	4 (3-11)	4 (3-7)	0.468**

* = Independent samples t test;

** = Mann-Whitney U test

Postoperative complications were observed in five patients in group 1 and three patients in group 2 (Table-3). The Clavien Grade 2 complications comprised blood transfusions, while the grade 3a complications included bladder catheterization by cystoscopy for one patient because the catheter was out of place and the need for percutaneous nephrostomy in one patient.

**Table 3:** Postoperative complication rates.

	Group 1 (n=20)	Group 2 (n=16)	p value
Complications, n, (%)	5 (25)	3 (18.75)	0.71
**Minor (Clavien 2)**	3	2	
Blood transfusion	3	2	
**Major (Clavien 3a)**	2	0	
Cystoscopy in operating room	1	0	
Percutaneous nephrostomy	1	0	

## DISCUSSION

Significant technological progress has been achieved in minimally invasive surgery with the use of Da Vinci^®^ robot and robotic surgery is successfully and reliably applied in the treatment of prostate cancer. However, the absence of tactile feedback and high costs appear to be disadvantages of this system. Since Binder et al. (5) and Menon et al. (12) performed robotic radical prostatectomy in 2000, the application of this technology has spread rapidly, and it is still growing. Its advantages, such as low perioperative blood loss, low postoperative pain, short hospital stay, and faster patient recovery, have made this technology more common (13).

Commonly accepted surgical principles include that the surgical anatomy needs to be dominated, dissection should be carried out in compliance with intraoperative variable dynamics and hemostasis and excretion should be carried out as required. The importance of these principles is even more prominent in laparoscopic surgeries. Errors can lead to increased complications, conversion to open surgery, and prolongation of the surgical time.

Concerning the learning curve of the robotic surgery, the research in the literature has concentrated on the console surgeon's performance. However, a successful robotic surgery can only be performed with a good team, and the patient - side surgeon is an important member of this team because she / he affects the safety and effectiveness of the operation (10). A mistake made by the patient - side surgeon may increase patient morbidity, negatively affect the surgical outcome, and even lead to open surgery (8). It has been reported in the literature that inexperienced patient - side surgeon can lead to the disappearance of suture needles and undesirable conditions such as major vessel injury (14, 15).

The bedside assistant plays a crucial role, especially in knotting of dorsal vein complex, retraction during bladder neck dissection, dissection of seminal vesicles, clipping of prostatic vascular pedicles, retraction during nerve preservation, delivery of sutures to the surgical field during anastomosis and specimen extraction (16). An experienced bedside assistant not only assists the case, but she / he also guides the console surgeon occasionally and can recommend steps that may facilitate the procedure.

In 2016, Potretzke et al. compared senior - level and junior - level bedside assistants in a robot assisted partial nephrectomy series with 414 patients. There were no differences in negative margin status, postoperative complications, estimated blood loss, or operative time between the groups. In their study, the console surgeons were experienced and, the research was carried out at a high - volume center; therefore, the bedside assistant may not have been a critical factor in the operations. In contrast, in our study, the console surgeons were both beginners, and the bedside assistant's.

Experince seems to have been an important component in the robotic surgery learning curve (17).

In our study, there was a significant difference between two groups with experienced and inexperienced bedside assistant in terms of trocar placement time, robot docking time, console time, specimen extraction time, anesthesia time and total operation time. Prostate volume, low surgical experience and lymph node dissection were reported to be factors that prolonged the surgical duration of the RARP procedure (18). In the present study, there was no difference in the rates of prostate volume and lymph node dissection among the groups. Often, the operation time is used to evaluate the surgeon's learning curve. As the experience of the surgeon increases, the surgery time shortens (19). Both surgeons in our study were in their learning curves; therefore, that the difference in the total operation times between the groups may depends on the bedside assistant's experience.

A prolonged surgery time in robot - assisted urological procedures may increase the risk of anesthesia exposure and side effects, perioperative complications like deep vein thrombosis and pulmonary embolism (20), postoperative complications (19) and positional injury (21). In a study evaluating RARP applied during the learning curve, D’Alonzo et al. reported that the anaesthesia and total surgery times were longer than those of open radical prostatectomy, and this led to a higher level of transient creatinine and increase in the use of intraoperative antihypertensive drugs (22). In addition, when the excessive Trendelenburg position is employed, facial, eyelid, conjunctiva and tongue edema can occur in patients. At the same time, soft tissue edema in the airway can delay the patient's extubation (23). Excessive Trendelenburg position and pneumoperitoneum affect the respiratory system by increasing arterial CO2 (24). Moreover, the increased risk of atelectasis due to decreased pulmonary compliance and functional residual capacity can lead to hypoxia (22). It has been shown that excessive Trendelenburg position may induce conjunctival edema and intraocular pressure, although it does not cause any problems later (25, 26).

Although there was no significant difference between the groups in terms of complications in our study, supporting the robotic surgical teams in the learning curve with an experienced bedside assistant may shorten the surgical time and reduce the risk of complications mentioned above. Patel et. al. reported that the operation time was less than 4 hours in the first 200 robotic series after the first 20 cases (27). In our study, the average operation time was less than 4 hours in the first 16 cases in the experienced bedside assistant group.

The surgical margin positivity is directly related to the quality of the surgery. In our study, surgical margin positivity was found to be consistent with the literature for both groups (7). There was no significant difference in this parameter between the two groups.

To the best of our knowledge, this study is the first study comparing the oncologic outcomes of RARP operations performed by two surgeons on the learning curve in the presence of experienced and inexperienced bedside assistants. Moreover, it represents one of the few studies in which the experience of bedside assistant was evaluated (17).

## CONCLUSIONS

The bedside assistant's experience in robotic surgeries may be viewed as an important factor when it comes to guiding the console surgeon with recommendations, increasing the cost effectiveness of operation and reducing the potential complications by shortening the operation time. This may be especially important in the learning curve for the console surgeon's initial cases.
